# A Background of a Volatile Plant Compound Alters Neural and Behavioral Responses to the Sex Pheromone Blend in a Moth

**DOI:** 10.3389/fphys.2017.00079

**Published:** 2017-02-10

**Authors:** Fabienne Dupuy, Angéla Rouyar, Nina Deisig, Thomas Bourgeois, Denis Limousin, Marie-Anne Wycke, Sylvia Anton, Michel Renou

**Affiliations:** Institut d'Ecologie et des Sciences de l'Environnement de Paris—ECOSENS, Institut National de la Recherche Agronomique-UPMCVersailles, France

**Keywords:** pheromone, odor background, interactions, volatile plant compounds, olfactory coding, behavior

## Abstract

Recognition of intra-specific olfactory signals within a complex environment of plant-related volatiles is crucial for reproduction in male moths. Sex pheromone information is detected by specific olfactory receptor neurons (Phe-ORNs), highly abundant on the male antenna. The information is then transmitted to the pheromone processing macroglomerular complex (MGC) within the primary olfactory center, the antennal lobe, where it is processed by local interneurons and projection neurons. Ultimately a behavioral response, orientation toward the pheromone source, is elicited. Volatile plant compounds (VPCs) are detected by other functional types of olfactory receptor neurons (ORNs) projecting in another area of the antennal lobe. However, Phe-ORNs also respond to some VPCs. Female-produced sex pheromones are emitted within a rich environment of VPCs, some of which have been shown to interfere with the detection and processing of sex pheromone information. As interference between the different odor sources might depend on the spatial and temporal features of the two types of stimuli, we investigated here behavioral and neuronal responses to a brief sex pheromone blend pulse in a VPC background as compared to a control background in the male noctuid moth *Agrotis ipsilon*. We observed male orientation behavior in a wind tunnel and recorded responses of Phe-ORNs and MGC neurons to a brief sex pheromone pulse within a background of individual VPCs. We also recorded the global input signal to the MGC using *in vivo* calcium imaging with the same stimulation protocol. We found that VPCs eliciting a response in Phe-ORNs and MGC neurons masked responses to the pheromone and decreased the contrast between background odor and the sex pheromone at both levels, whereas α-pinene did not interfere with first order processing. The calcium signal produced in response to a VPC background was tonic, lasting longer than the VPC stimulus duration, and masked entirely the pheromone response. One percent heptanal and linalool, in addition to the masking effect, caused a clear delay in responses of MGC neurons to the sex pheromone. Upwind flight toward the pheromone in a wind tunnel was also delayed but otherwise not altered by different doses of heptanal.

## Introduction

Olfaction is essential for insects to find a mate, a food source or an oviposition site. Volatile organic compounds released by their conspecifics, preys, or hosts are detected by olfactory receptor neurons (ORNs) housed in sensilla mainly situated on the insect antennae. The chemical tuning of the ORNs determines the range of volatile molecules that can be detected (Andersson et al., 2015). For insects flying toward odor sources not only the chemical composition, but also the concentration and dynamics of odor signals contain decisive information (Vickers, 2000, 2006; Cardé and Willis, 2008) and are transformed into spike firing patterns by the ORNs. This information about chemical signals is then transferred from the antennae to the primary olfactory centers, the antennal lobes (ALs). Thus, the raw input is progressively encoded by the olfactory system, enabling the insect to extract the ecologically relevant signals and to perform an adapted behavior. Insects communicating with pheromones for instance possess specialized olfactory receptor neurons (Phe-ORNs) each narrowly tuned to one of the pheromone components. It is generally admitted that these narrowly tuned olfactory receptors act as molecular filters preventing unspecific activation of the pheromone circuit by other odorants. Perception of pheromone is thus essentially achieved by a labeled line type of coding.

However, in their natural environment, insects are confronted with a rich olfactory world, which complicates the extraction of the relevant olfactory information. Terrestrial plants release a great variety of volatile organic compounds in the atmosphere. To illustrate the high diversity of volatile plant emissions, as many as 1,700 compounds have been inventoried within floral scents, and many of them provide behavioral cues to nectar foraging insects (Knudsen et al., 2006). The mixing ratios of volatile plant compounds (VPCs) in air are typically in the range of several ppb (Kesselmeier et al., 2000; Wiedenmyer et al., 2011). The effects of such large amounts of VPCs in the atmosphere on the perception of specific olfactory signals that are often released in much lower concentrations are still not fully understood. There is growing evidence that the chemical specificity of Phe-ORNs can be challenged by VPCs (Deisig et al., 2014; Renou et al., 2015). Different modes of interactions between pheromone components and VPCs have been reported. Ochieng et al. (2002) described synergy between linalool or (Z)-3-hexenol and the main pheromone component Z11-hexadecenal in the noctuid moth *Heliothis zea*, hypothesizing that the co-perception of pheromone and plant volatile could facilitate male detection of females. Synergy between pheromone and host plant signals has also been reported to occur within the macroglomerular complex in the ALs of *Cydia pomonella* (Trona et al., 2013). Supporting the hypothesis of plant volatiles acting as habitat cues facilitating mate location, in the field, males belonging to different moth species are attracted in greater numbers to traps baited with blends of pheromone and plant volatiles (Light et al., 1993; Landolt and Phillips, 1997; Deng et al., 2004), compared to traps baited with pheromone only. Enhancement of the number of oriented flights toward mixtures of pheromone with host-plant volatiles has also been observed under laboratory conditions in the wind tunnel (Schmidt-Büsser et al., 2009; Varela et al., 2011; Trona et al., 2013). In nectar-feeding insects, vegetative parts of a plant also provide an odor context that, added to floral volatiles, contributes to response specificity in flower selection (Riffell and Alarcon, 2013; Riffell et al., 2014). However, at Phe-ORN level, adding a VPC to the pheromone results more generally in a suppressive effect on pheromone response (Den Otter et al., 1978; Van Der Pers et al., 1980; Party et al., 2009), suggesting that a background of VPCs may constitute an odorant noise and thus decrease pheromone perception. Negative interactions have been described also at the behavioral level. For instance, host plant odor masking by non-host constitutive volatiles has been observed in silverleaf whiteflies resulting in negative interference with host plant colonization (Togni et al., 2010).

Rich sensory backgrounds have been repeatedly shown to affect signal extraction with significant impact on visual (Sasaki et al., 2006, 2008; Chen et al., 2014) or auditory communication (Brumm and Slabbekoorn, 2005; Chan et al., 2010; Schmidt and Römer, 2011; Siegert et al., 2013). Comparatively to these two sensory modalities that involve physical stimuli, the consequences of chemical backgrounds on the perception of olfactory signals are much less understood. The responses evoked in the locust sensory system by a foreground odorant vary when presented simultaneously, or after an ongoing background stimulus (Saha et al., 2013). In *Drosophila* larvae, the quality coding of odor components involves not only consistent and precise responses to a given compound in ORNs but also patterns of qualitatively variable responses by some other neurons, contributing to different degrees of activation in antennal lobe glomeruli and representing a key component of response variability in early olfactory processing (Hoare et al., 2008). The effects of VPCs on responses to the pheromone have been analyzed in individual ORNs, showing that besides altering qualitative and quantitative coding (Party et al., 2009; Rouyar et al., 2011) a VPC background increases response variability (Renou et al., 2015). It is thus particularly important to understand how insect olfactory systems code specific signals in an odor background, and challenging the moth pheromone system by VPCs provides an excellent study model.

In the present paper, using electrophysiological recordings and *in vivo* calcium imaging, we studied how a background of plant volatiles modifies the detection and early coding of the sex pheromone signal in Phe-ORNs and the macroglomerular complex (MGC), pheromone-specific part of the ALs, in the noctuid moth *Agrotis ipsilon*. Pheromone and plant odor processing, as well as interactions during simultaneous stimulation are well-investigated in this species, with well characterized ORN and antennal lobe neuron responses to both odor categories (Deisig et al., 2012 and references therein). We did not further analyze responses in so called ordinary glomeruli (OG), because earlier studies did not show any significant responses to the sex pheromone in the ordinary glomeruli in *A. ipsilon* and heptanal responses in OG were not affected by the pheromone (Deisig et al., 2012; Rouyar et al., 2015). Even though we are fully aware that a single VPC is not representative for a complex plant odor environment, we chose here, as a first step, to use individual VPCs, whose behavioral and physiological effects have been characterized previously in *A. ipsilon*. We further analyzed the upwind flight behavior of males toward mixtures of heptanal and the pheromone blend. The VPC heptanal used primarily in our study is released by various flowers, in particular linden flowers (*Tilia* sp.) that are highly attractive to adult *A. ipsilon* when foraging (Wynne et al., 1991; Zhu et al., 1993). We have recently shown that heptanal, although structurally very different from the three acetates that constitute the sex pheromone blend of *A. ipsilon*, activates the Phe-ORNs in this species (Rouyar et al., 2015). In the wind tunnel, male *A. ipsilon* are attracted by a linden flower extract (Deisig et al., 2012), but not by heptanal alone at the dose used (Rouyar et al., 2015). To determine if effects found for pheromone responses in a heptanal background are specific to this plant component, we additionally tested responses to the pheromone in backgrounds of two other plant volatiles.

## Materials and methods

### Insects

Larvae of *A. ipsilon* were reared in the laboratory on an artificial diet in individual plastic containers at 23°C and 60% relative humidity until their pupation. Sexes were separated at pupal stage, and females and males were kept in separate rooms under a reversed 16 h:8 h light:dark photoperiod under similar temperature and humidity conditions. Newly emerged adult moths were collected every day and provided *ad libitum* with a 20% sucrose solution. The day of emergence was considered day zero of adult life. Four or five day old sexually mature virgin males were used for electrophysiological, optical imaging and wind tunnel experiments. All experiments were performed during the scotophase, when male moths are sexually active.

### Chemicals

#### Sex pheromones

We used a synthetic sex pheromone blend based on the three components identified in natural extracts of the pheromone glands of female *A. ipsilon* (Picimbon et al., 1997; Gemeno and Haynes, 1998): (Z)-7-dodecen-1-yl acetate (Z7-12:OAc), (Z)-9-tetradecen-1-yl acetate (Z9-14:OAc) and (Z)-11-hexadecen-1-yl acetate (Z11-16:OAc), mixed at a ratio of 4:1:4. This blend was proven to be the most attractive to males in field tests (Causse et al., 1988) and it elicits similar behavior in a wind tunnel as natural extracts of the pheromone gland (Barrozo et al., 2010; Vitecek et al., 2013). We preferred to use the pheromone as a whole to investigate heptanal interactions with the complete stimulus at all integration levels, even though individual ORNs are known to respond each specifically to only one pheromone component (Jarriault et al., 2009). The three components were purchased from Sigma Aldrich (Saint-Quentin Fallavier, France) and diluted and mixed in hexane (>98% purity, CAS 110-54-3, Carlo-Erba, Val-de-Reuil, France). Doses of 10 ng and/or 100 ng of the sex pheromone blend were used in the experiments; these doses had previously been described as behaviorally and electrophysiologically active (Gadenne et al., 2001; Barrozo et al., 2010; Chaffiol et al., 2012; Deisig et al., 2012).

#### Volatile plant compounds

Heptanal (98% purity, CAS 111-71-7, confirmed by GC analysis, revealing no traces of pheromone compounds), racemic linalool (97% purity, CAS 78-70-6), and α-pinene (97% purity, CAS 80-56-8) were used for the experiments. Mineral oil (CAS 8042-47-5) was used to prepare volume-to-volume dilutions at 0.1 and 1%. All compounds were purchased from Sigma Aldrich (Sigma Aldrich, Saint-Quentin Fallavier, France).

### Olfactory stimulations

Odorants were delivered as described previously (Rouyar et al., 2011, 2015). Briefly, charcoal-filtered air was humidified and divided in eight equal flows (220 ml/min) directed each toward a three-way miniature valve. By activating the appropriate valve the flow could be directed to one of the 4 ml glass scintillation vials containing a stimulus source. Valves and vials were connected by PTFE tubing (1.32 mm ID) and hypodermic needles (18G size). To take into account differences in volatility and polarity, different types of stimulus sources were used for pheromone and heptanal or other VPCs. For the sex pheromone, a volume of the appropriate hexane solution was deposited into a section of PTFE tubing (1.6 mm ID; L = 20 mm) directly connected to a hypodermic needle inside the vial after solvent evaporation to constitute sources of 10 or 100 ng of pheromone blend. In turn, it would not be feasible to evaporate hexane without losing VPC, and the released amounts would rapidly decrease in time as the source exhausts. Thus, for VPCs, the scintillation vial contained 1 ml of solution in mineral oil, a non-volatile solvent, at the appropriate concentration vol/vol. Calibration data of our system are available from Party et al. (2009). Stimulus- and clean air-carrying tubes were assembled in a 10 cm long metal tubing constituting the “stimulation pencil.” A plastic cone of a P1000 pipette was placed at the output of the stimulation pencil to serve as a mixing chamber. The stimulation pencil was positioned ~5 mm in front of one antenna and focused on antennal sensilla when we recorded ORNs. In order to stimulate the whole antenna, the cone was placed 20 mm in front of the moth head in optical imaging experiments, or 5 mm in front of the antenna when we recorded MGC neurons intracellularly. Electric-valve programming was performed using a Valve Bank (AutoMate Scientific, Berkeley, USA) synchronized with the PC acquisition software.

### Electrophysiology

#### Single sensillum recording of ORNs

Male moths were briefly anesthetized with CO_2_ and restrained in a Styrofoam holder. One antenna was immobilized with adhesive tape. Single sensillum recordings were performed with electrolytically sharpened tungsten wires. The reference electrode was inserted into the antenna, 1–3 segments from the segment carrying the recorded sensilla. The recording electrode was inserted into the base of a long trichoid sensillum sampled along the length of an antennal branch. Four functional types of pheromone sensitive ORN types have been identified on *A. ipsilon* antennae (Renou et al., 1996; Gadenne et al., 1997). Previous investigations of their distribution along the antenna have shown that branch trichoid sensilla house almost exclusively the Phe-ORN type tuned very specifically to Z7-12:OAc. Z9-14:OAc tuned ORNs were found only at branch tips (Renou et al., 1996, Munoz, unpublished data); only two out of 100 neurons were found to respond to Z11-O16:Ac (Jarriault et al., 2010). Stimuli were presented at random and we retained only the firing of ORNs having responded to the 3-component blend over mineral oil background for further analysis. Due to their very similar response profiles, we analyzed responses of all active Phe-ORNs together. Recording and reference electrodes were connected to a Neurolog preamplifier (Digitimer, Hertfordshire, UK). The signal was filtered (0.2–10 kHz) and amplified 1000 times. The electrophysiological activity was sampled at 10 kHz and 12 bit resolution with a Data Translation DT3001 analog to digital card. Control of the acquisition board, spike sorting, and extraction of spike occurrence times were done using Awave software (Marion-Poll, 1995).

#### Intracellular recordings of MGC neurons

A male moth was slipped inside a 1 ml plastic pipette cone, cut off at the top, with its head protruding from the enlarged tip. The moth head was fixed with dental wax to prevent movements. After opening the head capsule, the brain was exposed by removing tracheal sacs and muscles (Gadenne and Anton, 2000). The neurolemma was removed from the surface of the antennal lobe to facilitate microelectrode penetration. Standard intracellular recording techniques with glass microelectrodes were used (Christensen and Hildebrand, 1987). The preparation was permanently superfused with Tucson Ringer (Christensen and Hildebrand, 1987). The reference electrode was placed in contact with the brain. A glass microelectrode was filled with 300 μM KCl and electrode resistances ranged from 20 to 100 MΩ. The electrode was inserted randomly in the MGC area of the AL until intracellular contact with a neuron was established. Penetrating neurons within the MGC area results in a vast majority of neurons showing an excitatory response to the pheromone followed by an inhibition phase in *A. ipsilon*. Neurons with this type of response pattern have previously been identified as projection neurons through intracellular staining (Jarriault et al., 2009; Barrozo et al., 2011; Chaffiol et al., 2012). Electrical signals were amplified with an AxoClamp-2B amplifier (Molecular Devices, Sunnyvale, California, USA). Neural activity was recorded, digitized, and spike occurrence times extracted using P-clamp software (Molecular Devices, Sunnyvale, California, USA).

#### Experimental protocol

**Figure 1:**
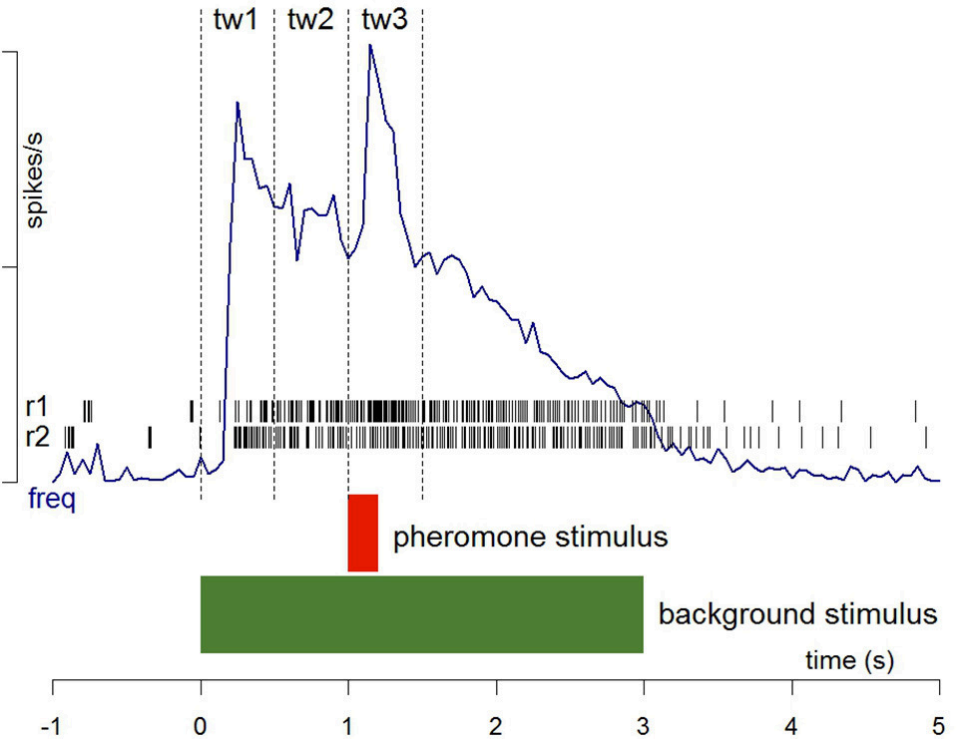
**Stimulation protocol and procedures for the analysis of spike firing**. Two recording samples from Phe-ORNs are shown as raster plots (r1 and r2). Frequency plots (freq) were averaged from the firing activity of several individual neurons recorded continuously before, during and after stimulation with the two stimuli. The limits of the three time windows (0.5 s each) used to measure activity in response to background presentation (tw1), before pheromone presentation (tw2) and during pheromone presentation (tw3) are indicated. The relative response to pheromone was calculated as (tw3-tw2). See Section Materials and Methods for details.

We compared the responses of Phe-ORNs and MGC neurons to the pheromone blend in a mineral oil background with responses to the pheromone in a background of heptanal, linalool or α-pinene (diluted in mineral oil) by stimulating the antenna with a 3-s long pulse and adding a 200 ms pulse of pheromone at a dose of 10 and 100 ng during the background stimulation (Figure 1). The pheromone pulse occurred 1 s after the onset of the background. Two concentrations of heptanal background, 0.1 and 1%, 1% linalool and 1% α-pinene were tested. Responses to the VPC background alone (without a pheromone pulse) were also recorded. Phe-ORNs were recorded during 30 s before starting the stimulation program and 30 s after, i.e., with inter-stimulus intervals of 60 s. Due to a lower stability of intracellular recordings, it was necessary to shorten the procedures for MGC neurons. Thus, odorant stimulation started 5 s after recording onset and inter-stimulus-intervals lasted for 10 s.

### Calcium imaging

A male moth was fixed in a plastic recording chamber and the head was opened to allow free access to the ALs. For staining, 10 μl of dye solution (Calcium Green 2-AM dissolved in Pluronic F-127, 20% in DMSO, Molecular Probes, Eugene, OR, USA, diluted in saline solution) were bath-applied on each brain for a minimum of 1 h. Recordings were obtained with a T.I.L.L. Photonics imaging system (Martinsried, Germany) coupled to an epifluorescent microscope (BX-51WI, Olympus, Hamburg, Germany) equipped with a 10x (NA 0.3) water immersion objective. Series of images were recorded with a 14-bit monochrome CCD camera (Andor iXON, cooled to -70°C, image size 1004 × 1002 pixels, binning on chip: 4). Excitation light was applied using a monochromator (T.I.L.L. Polychrom V) while the microscope was equipped with a GFP-BP filter set composed of a 490 nm dichroic beamsplitter and a 525/550 nm emission filter.

Each animal was subjected to up to three series of 15 olfactory stimuli with inter-stimulus intervals (ISIs) of 100 s to adapt to the slow dynamics of the fluorescence signal, and one AL was recorded in each insect. The stimulation protocol was the same as in single sensillum and intracellular recordings, however, α-pinene was not tested as background odor due to a limited number of possible stimulations to be administered. Each recording lasted for 20 s, with the background odor stimulation starting 3 s after recording onset and lasting for 3 s. Short pheromone pulses (200 ms pulses) were given 4 s after recording onset.

Raw data were treated for reduction of photon (shot) noise using custom-made software written in IDL (Research Systems Inc., Colorado, USA) and signal time courses were illustrated with Visual Basic (Microsoft Excel). Relative fluorescence changes (ΔF/F) were calculated as (F - F_0_)/F_0_, with a reference background F_0_ 1–2 s before odor stimulation and F at signal maximum (about 6 s after recording onset, i.e., 3 s after background odor- and 2 s after pheromone stimulation-onset). For each identified activity spot, the time course of relative fluorescence changes was calculated by averaging 25 pixels (5 × 5) at the center of each activity spot and well within its borders. The amplitude of odor-induced responses was calculated as the mean of three frames at the signal's maximum (frames 29–31) minus the mean of three frames before the stimulus (frames 7–9). This value was used in all computations. Activity maps were obtained by calculating the signal difference between the max and min signal amplitude (6 and 12 s after recording onset).

### Wind tunnel experiments

Male moths' flight behavior in response to the pheromone blend alone or in a background of heptanal was observed in a Plexiglas wind tunnel measuring 190 cm length × 75 cm width × 75 cm height (VT Plastics, Genevilliers, France). Both ends of the tunnel were enclosed with white synthetic fabric allowing air to pass through but prevented insects from escaping. Air movement was provided by an exhaust fan at the downwind end of the tunnel sucking the air at a speed of 0.3 ms^−1^ and evacuating odorized air out of the building. The tunnel was maintained in dark except for one red visible light source allowing visual observations and side infrared illumination for video tracking provided by an array of eight 5 W IR lamps, of 54 LEDs each, emitting at 850 nm. Randomly arranged patterns of 10 cm diameter black circles were positioned 30 cm behind the rear wall of the tunnel to provide visual cues to the moths.

Moth flight tracks were recorded and analyzed using Trackit 3D 2.0 (SciTracks, Pfaffhausen, Switzerland). Two cameras (Basler Pilot, piA640–210 m with Tamron ½” 4-12F/1.2 lenses) were positioned above the tunnel at 60 cm from each other to cover the whole tunnel flight section with overlapping fields. Images from the two cameras were analyzed in real time and the x, y, and z coordinates of moth's position were extracted every 10 ms. Tracks were stored in form of “.csv” files.

Experiments were performed at 23°C, 40 ± 10% relative humidity, during the second half of the scotophase (i.e., 4–7 h after lights turned off) which corresponds to the peak activity of male *A. ipsilon*. A single 5-day old virgin male was placed on a 36 cm high platform in the middle of the tunnel width and 160 cm downwind from the odor source. After allowing the moth a 1-min time of adaptation to the tunnel environment, we applied the odor stimulation and monitored its behavior for 3 min. We compared responses to either the pheromone at 100 ng alone, or with heptanal at 0.1 or 1% dilutions in mineral oil. Control experiments (no odor) were performed with a clean filter paper as source. Each individual was tested only once. Olfactory stimuli were delivered using the same stimulator as in electrophysiological experiments. Hypodermic 18G needles were used as odor nozzles delivering odorized air flows at the center of the tunnel upwind end. Sex pheromone blend diluted in hexane was deposited on a filter paper introduced in a 4 ml scintillation vial after solvent evaporation. Heptanal was diluted in 1 ml of mineral oil.

Three behavioral items were scored during an observation period of 180 s: take-off, partial flight (flight half way between the release site and the odor source) and source flight (arrival within 20 cm of the source). All males stimulated with the pheromone blend showed activation and performed take-off in <90 s after test onset, thus 90 s was taken as the time limit for scoring these two items for all subsequent experiments. Take-off times were also measured to calculate response delay after onset of pheromone stimulation.

### Statistical analyses

For electrophysiological experiments, spike occurrence times were analyzed using custom-written R scripts (R Core Team, 2013). Firing rates were calculated using the local slope of the cumulative function of spike times (Blejec, 2005) calculated over a moving spike window between the n-2 and n+2 spikes (5 spikes). Thus, each spike was attributed a firing rate and its occurrence time. To quantify and compare the response intensity of receptor and central neurons with very different firing patterns, we measured the maximum firing rate. It reflects very well fast changes in neuron activity. It occurs early in the response course, and can be measured whatever the response shape. Its intensity can be determined independently from latency and its level can be used to evaluate signal salience. The maximum firing rate was determined during three critical time windows tw1: background onset = 0–0.5 s; tw2: level of activity before pheromone pulse = 0.5–1 s; tw3: response to pheromone pulse = 1–1.5 s (Figure 1). The mean ± standard error of the maximum firing rates was calculated for each stimulation. Data were compared using a Student *t*-test for paired data followed by tests to check for data set normality (Shapiro test) and variance homogeneity (Fisher-Snedecor test), in the case of ORN recordings, or were compared using a Wilcoxon test for paired data for MGC neuron recordings. To analyze the dynamic of the response of central neurons, we plotted the Kaplan-Meier estimator using the time of occurrence of the maximum firing rate (peak) as a variable.

Calcium responses induced by different odors in different glomeruli were compared using Statistica (Version'99, http://www.statsoft.com). We performed 1- or 2-way ANOVAs for repeated measures with the two factors odor and glomerulus. When interactions among factors were significant, simple effects were analyzed by means of a 1-way ANOVA with or without the RM factor, and then followed by a Tukey's test for *post-hoc* comparisons if necessary.

For wind tunnel experiments, a Fisher's exact test was used to compare scores of response of male moths to heptanal and the pheromone.

## Results

### VPC activation masks pheromone responses in Phe-ORNs

**Figure 2:**
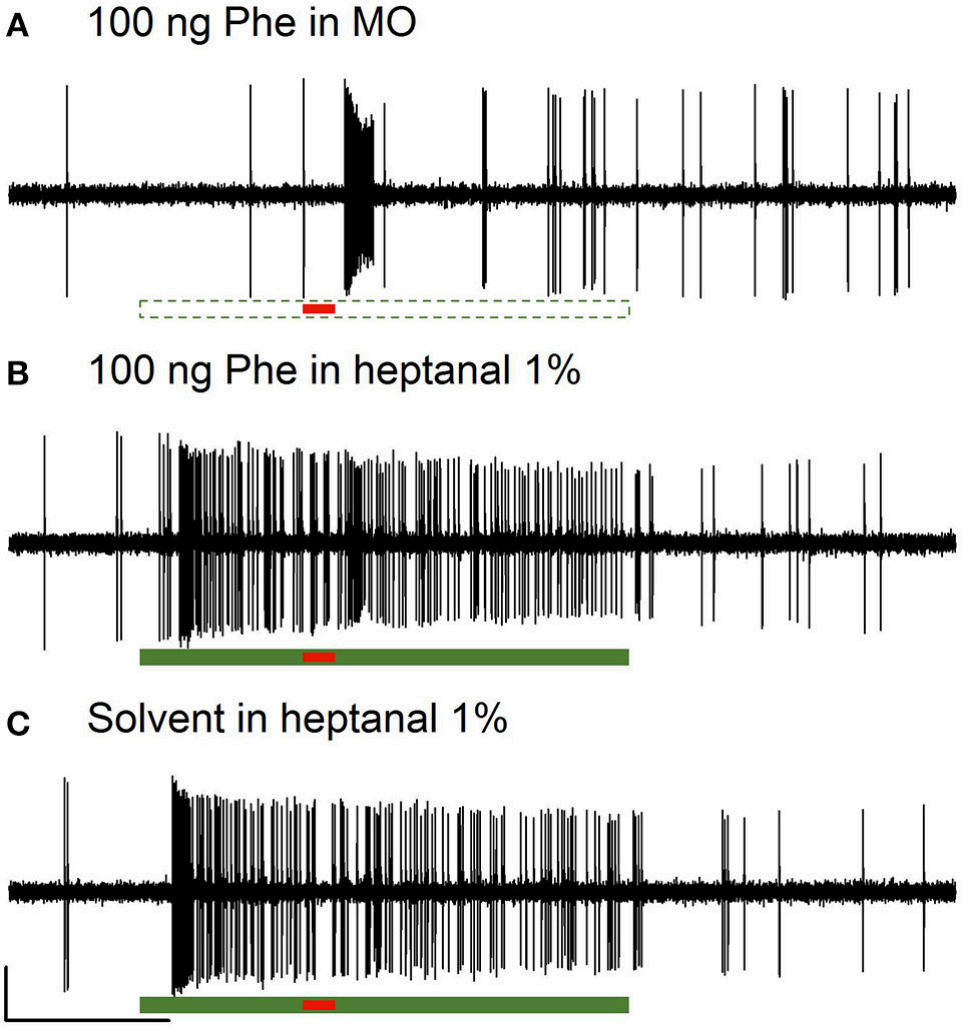
**Responses of Phe-ORNs to pheromone stimulation in a VPC background in male ***Agrotis ipsilon*****. Typical examples of extracellular recordings obtained from long trichoid sensilla housing the Phe-ORNs. The Phe-ORNs responded to a short pulse of the pheromone blend (Z7-12:OAc, Z9-14:OAc, Z11-16:OAc at a 4:1:4 ratio, 100 ng) **(A)** but when heptanal was presented as a background the response was masked **(B)** by the firing activity triggered by heptanal itself **(C)**. Scale: vertical bar = 1 mV; horizontal bar = 1 s. The short horizontal red bar underneath the recordings indicates the pheromone stimulation (0.2 s, **A,B**) or a solvent presentation **(C)**. The long horizontal green bars indicate the presentation of mineral oil (dashed, empty bar, **A**) as a control or heptanal (solid bar, **B,C**, 3 s).

**Figure 3:**
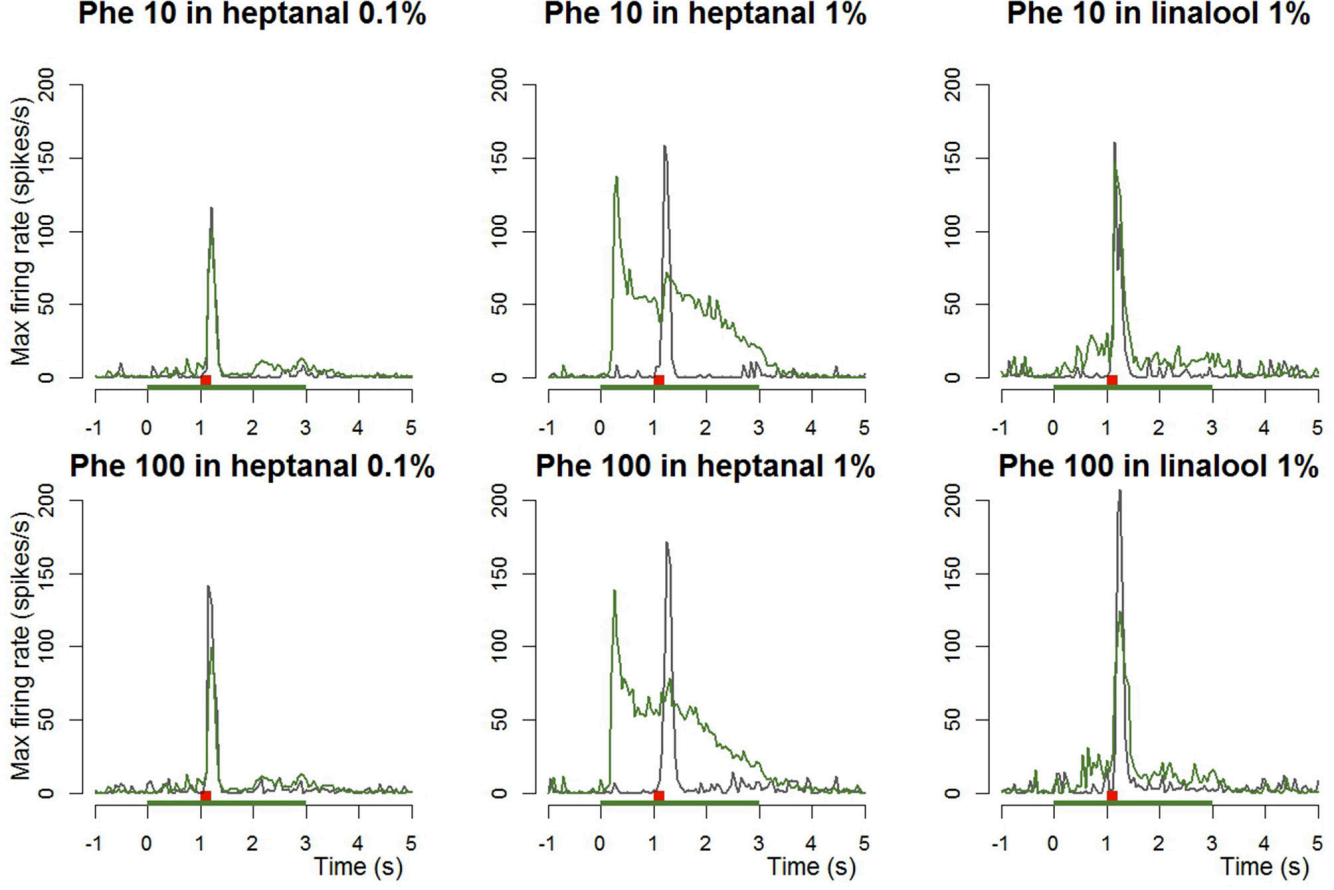
**Dynamic of the responses of Phe-ORNs to a pheromone pulse in different backgrounds**. Frequency plots show maximum firing frequency per 50 ms bins averaged from *n* = 14–20 neurons. Either 10 or 100 ng of pheromone was given as a short pulse (0.2 s, red bars) in air (gray curves) or in a background of VPCs (heptanal 0.1 and 1%, linalool 1%, green bars; green curves).

The Phe-ORNs of *A. ipsilon* responded to short pulses of the pheromone blend (Z7-12:OAc, Z9-14:OAc, and Z11-16:OAc in a 4:1:4 ratio) at the low (10 ng) and high (100 ng) doses by a fast and phasic increase of the firing (Figures 2A, 3).

**Figure 4:**
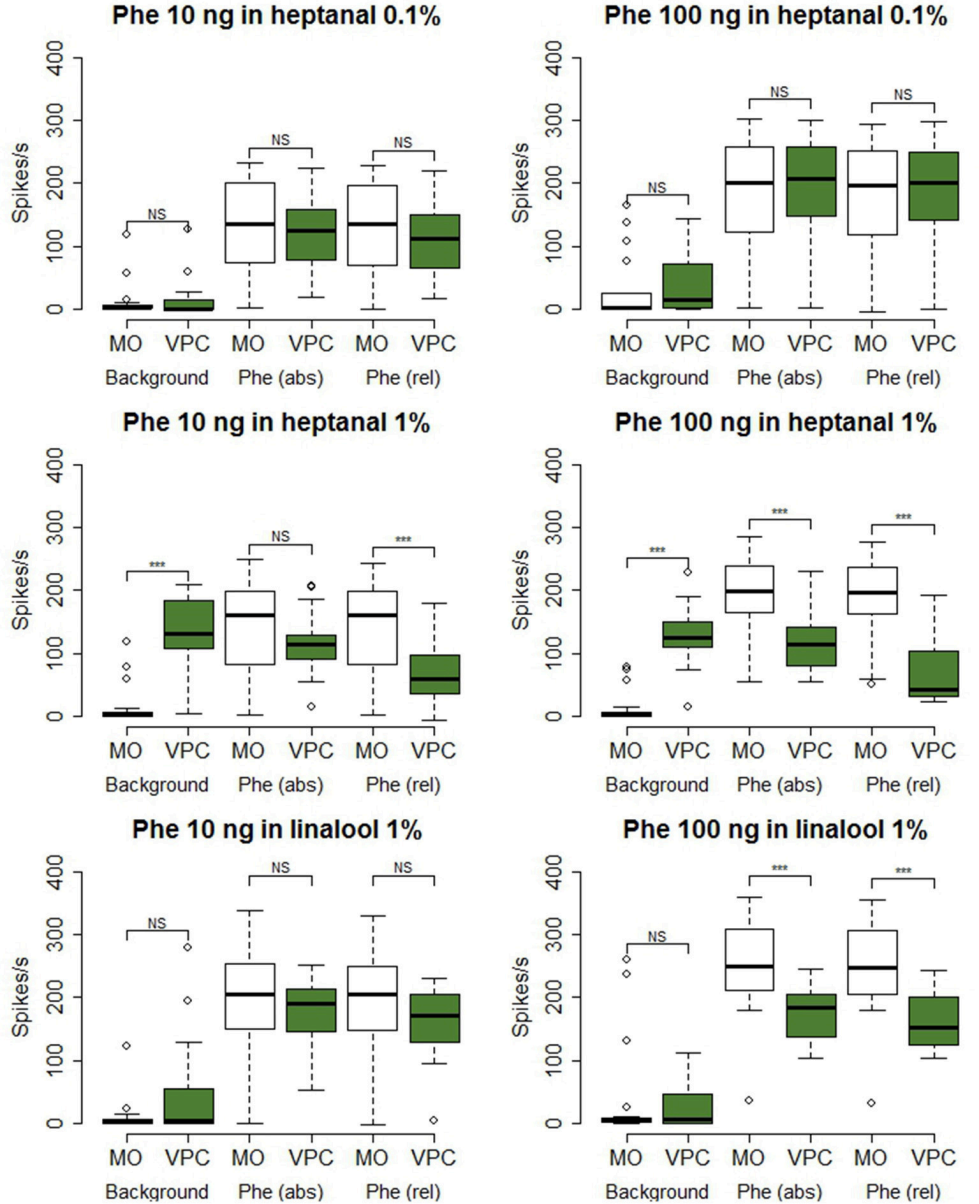
**Statistical analysis of Phe-ORN responses to pheromone stimulation in a VPC background**. The box plots show Phe-ORN responses to the different backgrounds of VPCs (measured as the maximum firing rate at tw1, from 0 to 0.5 s after background onset, see Figure 1, the absolute response (abs) to the pheromone pulse (maximum firing rate at tw3 from 1 to 1.5 s), and the relative response (rel) to the pheromone pulse (maximum firing rate reached during time window tw3 (1–1.5) minus the mean firing rate immediately before the pheromone pulse (tw2: 0.5–1 s). White bars represent the responses in control background (pure mineral oil). Green bars represent the odorized backgrounds (VPCs, diluted in mineral oil). The lower whisker presents the minimum, the lower hinge of the box the first quartile, the line inside the box the median, the upper hinge the third quartile, and the extreme of the upper whisker the maximum; outlier values are indicated by a circle. *N* = 17 (heptanal 0.1%), 19 (heptanal 1%), or 19 (linalool). Asterisks indicate significance level: ^***^p < 0.001; NS not significant; Wilcoxon test for paired data.

Phe-ORNs did not respond to the low level (0.1%) of heptanal background (mean and standard deviation of the maximum firing rate 9.81 ± 22.1, *N* = 34; labeled “Background” in Figure 4). In turn, a high level (1%) of heptanal elicited a phasic-tonic response (Figure 2C) with a maximum firing rate of 132.9 ± 50.7 spikes/s (mean of *N* = 44 recordings) at heptanal onset (see “Background” in Figure 4). This increase in firing due to heptanal 1% relatively to mineral oil was highly significant (V = 17, *p* = 1.171*10^−7^ and V = 7, *p* = 3.03*10^−9^). After 1 s (corresponding to the tw2 as shown in Figure 1) in heptanal 1% the level of firing was still very high (110.9 ± 40.8 spikes/s).

When presenting a combination of both stimuli, the responses to 10 or 100 ng of the pheromone blend were unaffected by heptanal 0.1% (Figure 3) and the statistical analysis confirmed the absence of significant differences between relative responses to the pheromone pulse in control *vs*. heptanal 0.1% odorized background (121.2 ± 62.4 vs. 129 ± 80.7 spikes/s in non-odorized air, *N* = 17 for 10 ng of pheromone, V = 160, *p* = 0.610; 192.1 ± 80.7 vs. 173 ± 104.8, *N* = 17 for 100 ng of pheromone, V = 138, *p* = 0.838—“Phe (abs)” in Figure 4).

In heptanal 1%, the high levels of firing measured during the pheromone pulse were not significantly different between the two doses of pheromone (Figures 3, 4; 117.2 ± 48.9 at 10 ng, 120.3 ± 50.5 at 100 ng, means and standard deviation of *N* = 19–25 recordings, V = 241, *p* = 0.537). Furthermore, the relative activity obtained after deducing the level of firing immediately before the pheromone pulse was considerably decreased, compared to MO [“Phe (rel)” in Figure 4, V = 353, *p* = 0.0056 and V = 575, *p* = 1.9*10^−8^ for 10 and 100 ng of pheromone, respectively]. Thus, the activity triggered by heptanal at 1% masked the ORN response to the pheromone pulse in most of the recordings (see example in Figure 2B), and altered intensity coding of pheromone concentration.

Compared to heptanal, linalool at 1% triggered a smaller but significant increase in the firing of Phe-ORNs (38.0 ± 63.6 at linalool onset vs. 9.1 ± 18.8 spikes/s, mean of *N* = 37, V = 190, *p* = 0.034). The linalool background did, however, not significantly alter the response to the 10 ng pheromone blend (V = 80, *p* = 0.560), but it significantly reduced the maximum firing rate in response to 100 ng pheromone blend (252.1 ± 75.5 spikes/s in non-odorized air vs. 177.4 ± 44.4, V = 12, *p* = 0.0003, *N* = 19; Figures 3, 4). The relative response to pheromone (contrast) was reduced for the higher pheromone dose (100 ng: W = 312, *p* = 4.95*10^−5^) but the difference was not significant for 10 ng (W = 218, *p* = 0.28; Figure 4).

### VPC background increases pheromone-elicited Ca^2+^ signals in the MGC

**Figure 5:**
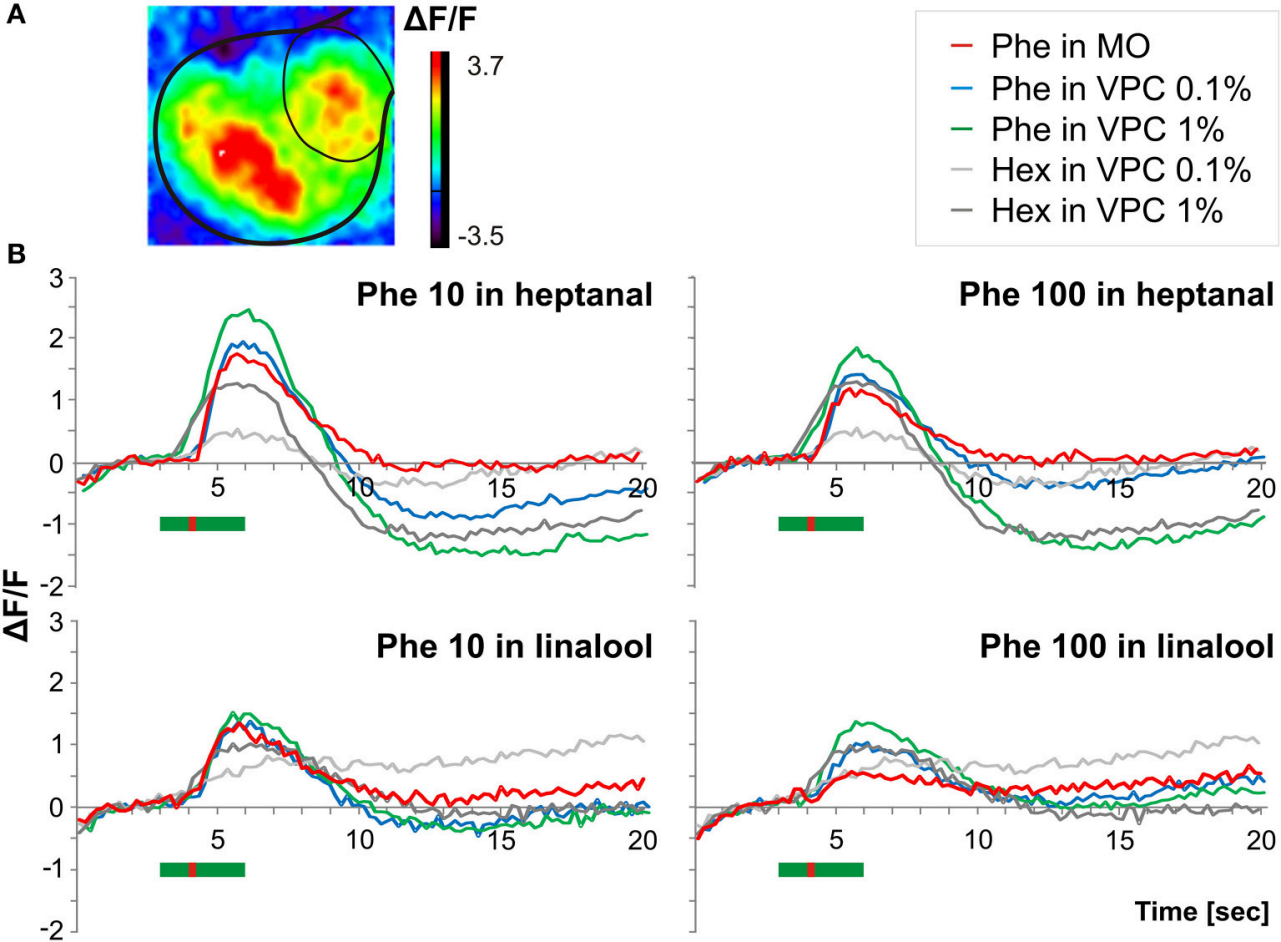
**Pheromone-induced Ca^**2+**^ signals within the MGC in a VPC background (A)** Odor induced *in vivo* calcium imaging signals obtained in a male stimulated with a heptanal background in which a short pulse of pheromone was added **(B)** Time courses of odor-evoked calcium activity (% change in fluorescence) in the MGC in response to pulses of the pheromone blend (10 and 100 ng) applied during a long-lasting (3 s) background of the two VPCs heptanal (*n* = 10) and linalool (*n* = 6) at two doses (0.1 and 1%). The green bar in **(B)** indicates the background stimulus (3 s) while the red bar indicates the pheromone pulse (200 ms Phe, Pheromone; Min Oil, Mineral oil; Hex, Hexane, VPC, volatile plant compound.

Short pheromone pulses at low (10 ng) and high (100 ng) doses activated the MGC (Figure 5B, red curves; *N* = 10 moths). Response delays in the MGC were similar to those observed for Phe-ORNs (Figures 5B, red curves; *N* = 10 moths). Interestingly, the weakest pheromone pulse (10 ng) induced significantly larger responses than the strongest pulse [100 ng; 1-way ANOVA fixed effects *F*_(1, 9)_ = 22.35, *p* = 0.001]. Heptanal backgrounds alone also elicited a long-lasting and dose-dependent increase in fluorescence within the MGC [1-way ANOVA fixed effects *F*_(1, 9)_ = 11.03, *p* = 0.009; light gray and dark gray curves in Figure 5B]. The duration of responses to heptanal were, however, longer than for the pheromone.

When presenting pheromone pulses during VPC backgrounds, the fluorescence increases largely overlapped each other in spite of the 1-s shift between the two stimulus onsets. Thus, we could not distinguish and measure the contribution of heptanal and pheromone separately in the global Ca^2+^ signal (Figure 5B, blue and green curves). We thus compared the maximum level of fluorescence reached during presentation of the combined stimuli (see example in Figure 5A). While a heptanal 0.1% background did not change responses to 10 ng of pheromone [Figure 5B, upper left panel, red vs. blue curve: 1-way ANOVA fixed effects *F*_(1, 9)_ = 1.35, *p* = 0.275], thus showing hypoadditivity, it did increase significantly the response to 100 ng of pheromone [additivity, Figure 5B, upper right panel, red vs. blue curve: 1-way ANOVA fixed effects *F*_(1, 9)_ = 10.27, *p* = 0.01]. In turn, additivity was observed with heptanal 1% background both for 10 ng pheromone [Figure 5B, upper panels, green vs. red curves: 1-way ANOVA fixed effects *F*_(1, 9)_ = 6.64, *p* = 0.029], and 100 ng pheromone [1-way ANOVA fixed effects *F*_(1, 9)_ = 10.35, *p* = 0.01].

With linalool as background (*N* = 6), responses to 10 and 100 ng of pheromone were globally weaker, compared to the heptanal background group (*N* = 10). Again, the low dose (10 ng) of pheromone induced significantly stronger calcium responses in the MGC compared to 100 ng [1-way ANOVA fixed effects *F*_(1, 5)_ = 49.82, *p* = 0.0009]. Both levels of linalool background led to similar increases in fluorescence in the MGC [Figure 5B, lower panels bright vs. dark gray lines, *N* = 6, *F*_(1, 5)_ = 2.69, *p* = 0.162]. Neither 0.1% nor 1% linalool background modified the responses to 10 ng of the pheromone [Figure 5B, lower left panel, red vs. blue curve: *F*_(1, 5)_ = 2.16, *p* = 0.201; *F*_(1, 5)_ = 5.12, *p* = 0.073, respectively]. In turn, additivity was observed with 100 ng pheromone: signals to 100 ng of the pheromone in linalool being significantly stronger compared to pheromone only both in 0.1% [Figure 5B, lower right panel, red vs. blue curve: *F*_(1, 5)_ = 27.30, *p* = 0.003] and l% linalool backgrounds [1 *F*_(1, 5)_ = 70.69, *p* = 0.0004]. Calcium imaging thus revealed no effect or enhancement on fluorescence levels during presentation of pheromone stimuli in VPC backgrounds.

### VPC activation of MGC neurons masks pheromone responses

**Figure 6:**
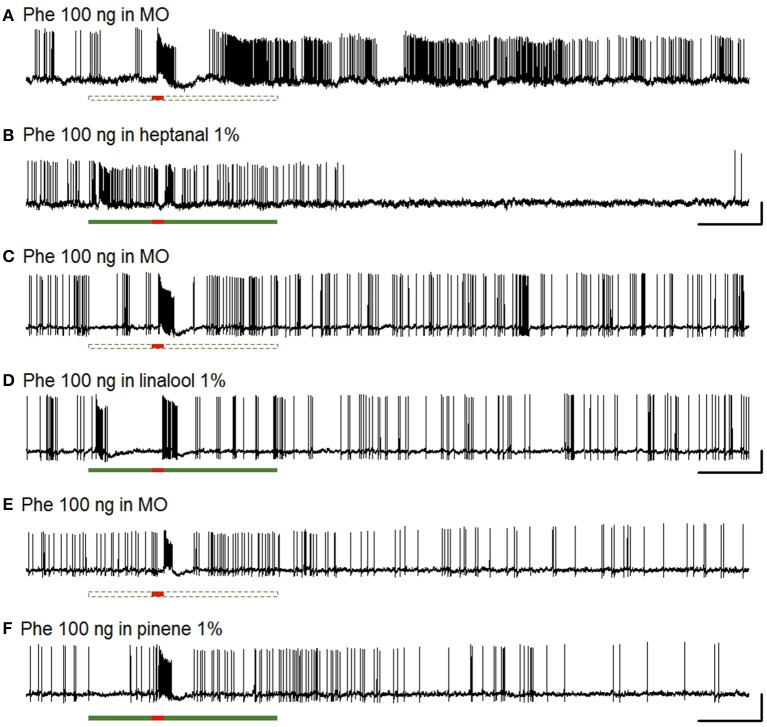
**Responses of MGC neurons to pheromone stimulation in a VPC background**. Typical examples of intracellular recordings obtained from pheromone-responding MGC neurons. The MGC neurons exhibited a multiphasic response to a short pulse of the pheromone blend (100 ng) **(A,C,E)** and responded also to a long presentation of heptanal **(B)** and linalool **(D)**, but not α-pinene **(F)**. Scale: vertical bar = 10 mV; horizontal bar = 1 s.

The pheromone blend elicited a multiphasic response in the large majority of the antennal lobe neurons recorded in the MGC, independently of the presence of a VPC background. The pheromone responses were characterized by an initial excitation, followed by an inhibition and in some cases by a long-lasting second excitatory phase (Figures 6A,C,E). This pattern is similar to that of type A neurons described earlier (Chaffiol et al., 2012).

**Figure 7:**
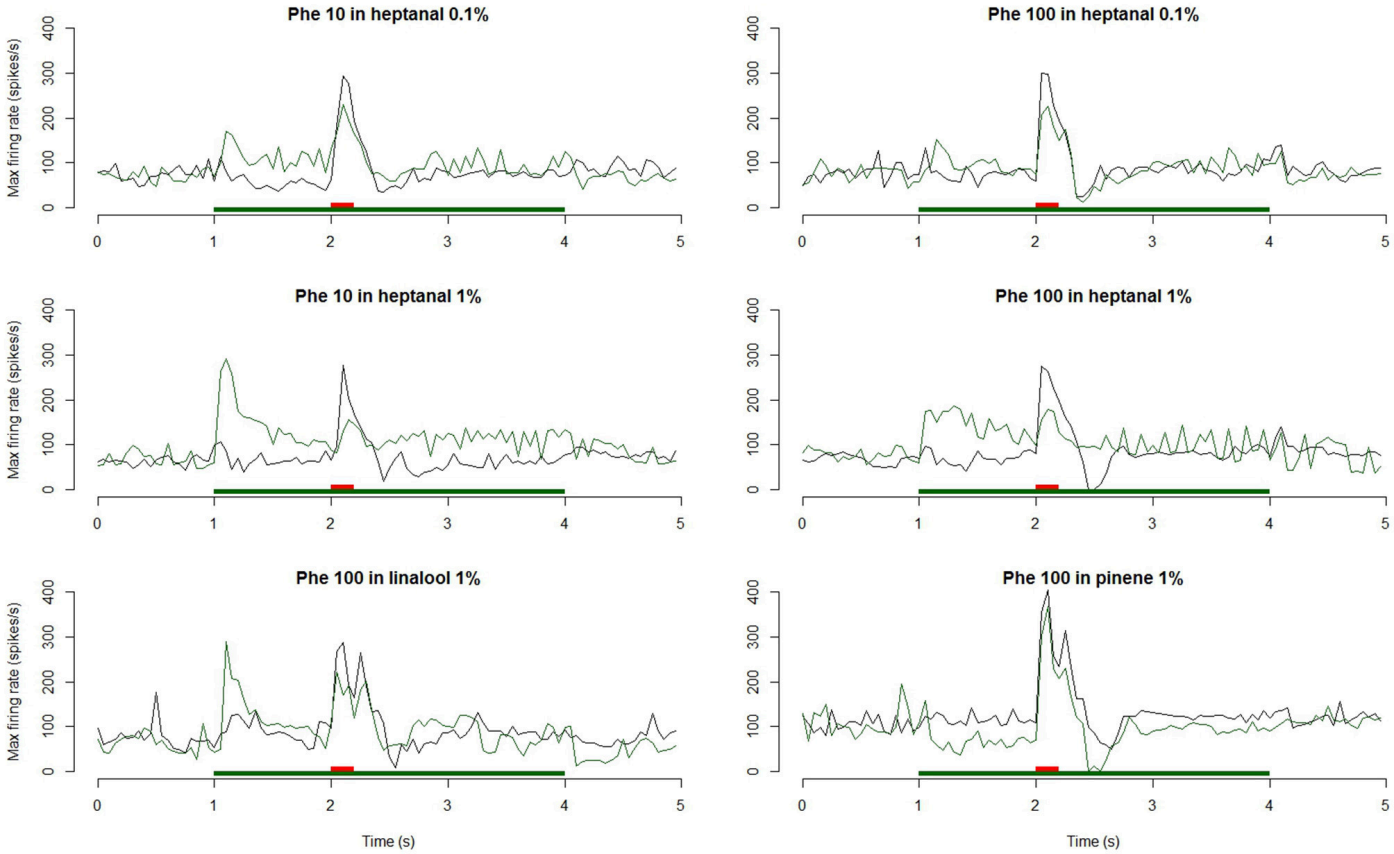
**Dynamic of the responses of MGC neurons to a pheromone pulse in different backgrounds**. Frequency plots show the responses of MGC neurons to a pheromone pulse (10 or 100 ng—red bars) in air or in a background of VPCs (heptanal 0.1 and 1%, linalool 1%, α-pinene 1%—green bars). Plots are averaged from *n* = 24 (Phe 10 ng—Heptanal 0.1%), 16 (Phe 100 ng—Heptanal 0.1%), 36 (Phe 10 ng—Heptanal 1%), 28 (Phe 100 ng—Heptanal 1%), 28 (Phe 100 ng—α-Pinene 1%), or 22 (Phe 100 ng—linalool 1%) recordings. The responses to the pheromone were masked by 1% heptanal and linalool and only at the 100 ng pheromone dose by 0.1% heptanal.

**Figure 8:**
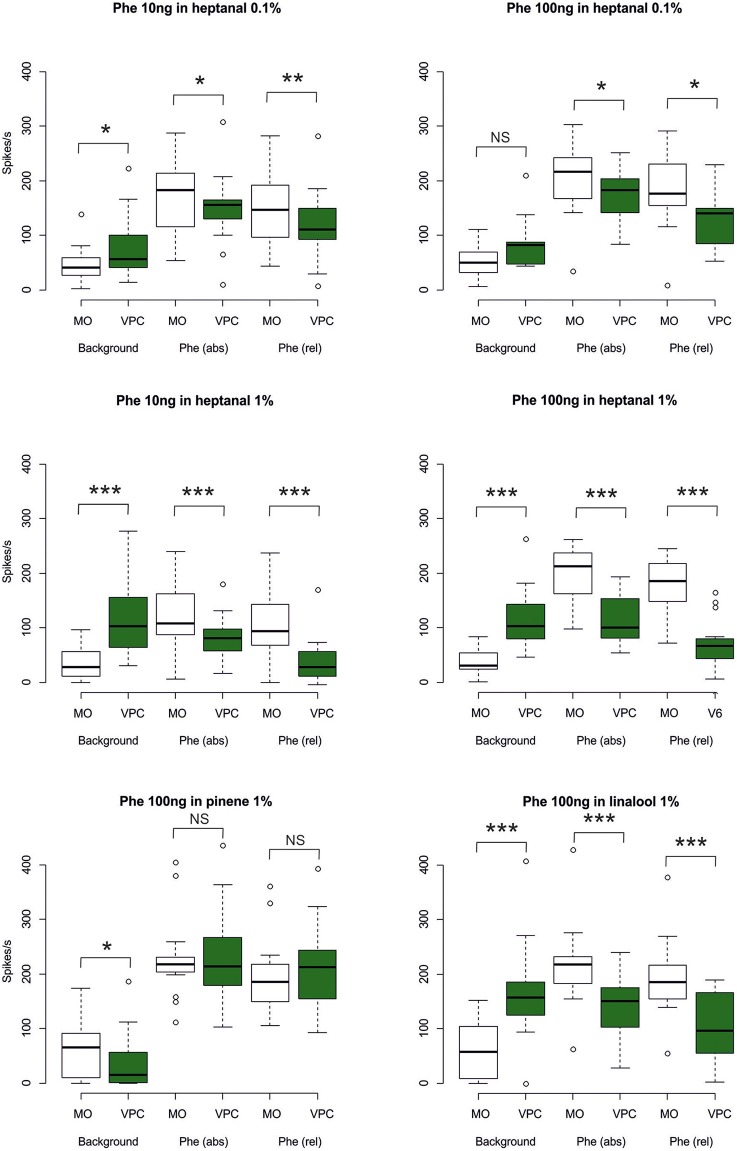
**Statistical analysis of MGC neuron responses to pheromone stimulation in a VPC background**. The box plots show MGC neuron responses to the different backgrounds of VPCs during a time window of 0.5 s (maximum firing rate at tw1, see Figure 1), the absolute response (abs) to the pheromone pulse (maximum firing rate at tw3), and the relative response (rel) to the pheromone pulse (maximum firing rate reached during tw3) minus the mean firing rate immediately before the pheromone pulse (tw2). White bars represent the control background (pure mineral oil). Green bars represent the odorized backgrounds (VPCs, diluted in mineral oil). The lower whisker presents the minimum, the lower hinge of the box the first quartile, the line inside the box the median, the upper hinge the third quartile, and the extreme of the upper whisker the maximum; outlier values are indicated by a circle. *N* = 17 (Phe 10 ng in heptanal 0.1%), 10 (Phe 100 ng in heptanal 0.1%), 22 (Phe 10 ng in heptanal 1%), 16 (Phe 100 ng in heptanal 1%), 14 (Phe100 ng in linalool), 15 (Phe 100 ng in α-pinene). Asterisks indicate significance level: ^*^p < 0.05, ^**^p < 0.01, ^***^p < 0.001; NS not significant; Wilcoxon test for paired data.

A heptanal 0.1% background evoked a small but significant response in MGC neurons only in certain series of experiments (Figure 7). In turn, heptanal at 1% consistently lead to phasic-tonic responses in MGC neurons (V = 1, *p* = 9.5*10^−7^ and V = 0, *p* = 3.5*10^−5^), as did linalool at 1% (V = 0, *p* = 0.0017; Figures 7, 8). These long stimulations with plant volatiles elicited responses showing highly variable patterns of firing activity. Indeed, tonic, phasic-tonic, mono-phasic, and multiphasic responses were observed. Moreover, none of these patterns occurred more often than the others (data not shown).

The heptanal background at both concentrations and the linalool background at 1% decreased the absolute response frequency to the pheromone significantly as compared to the MO background (V = 125, *p* = 0.02 and V = 47, *p* = 0.049 for heptanal 0.1%, V = 233, *p* = 1.8*10^−4^ and V = 136, *p* = 3*10^−5^ for heptanal 1%, V = 99, *p* = 0.0017 for linalool; Figures 6B,D, 7, 8). Also the relative increase in firing activity over the level of heptanal-induced activity at presentation of the pheromone pulse was significantly reduced (V = 132, *p* = 0.0066 and V = 48, *p* = 0.04 for heptanal 0.1%, V = 247, *p* = 6.7*10^−6^ and V = 136, *p* = 3*10^−5^ for heptanal 1%, V = 105, *p* = 1.2*10^−4^ for linalool; Figure 7). Thus, heptanal and linalool had a masking effect on MGC neuron responses to pheromone.

Differently from heptanal and linalool, a background stimulation with α-pinene at 1% significantly decreased the firing activity of MGC neurons, but did not affect the responses to the pheromone pulse (Figures 6F, 7, 8).

**Figure 9:**
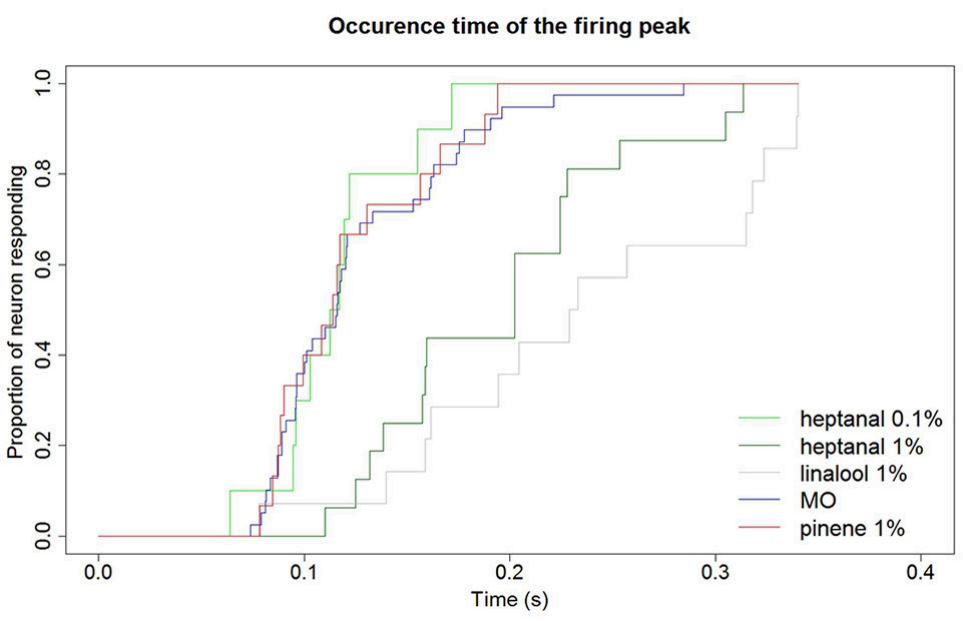
**Delay of MGC neuron responses to pheromone in different backgrounds**. Backgrounds of heptanal and linalool at 1% slow down the response of MGC neurons to the pheromone compared to a mineral oil background. Response dynamics is not altered by heptanal at 0.1% and α-pinene at 1%. Kaplan Meier estimator curves showing the time at which the maximum firing frequency is reached in response to the 100 ng pheromone blend in different backgrounds. MO (*N* = 43), heptanal 0.1% (*N* = 20), heptanal 1% (*N* = 32), linalool 1% (*N* = 14), α-pinene 1% (*N* = 15).

In addition to a reduction of the maximum firing frequency, a background of heptanal and linalool at 1% also delayed the occurrence of the peak frequency in MGC neurons (Figure 9). In turn, α-pinene at 1% did not modify response dynamics.

### Wind tunnel

The behavior of males in the wind tunnel was not different in presence of heptanal 0.1% alone, compared to the solvent, mineral oil (Control, Table). As many male moths were activated in response to the pheromone in presence of heptanal 0.1% (χ^2^ = 0.0, df = 1, *p* = 1.0), as in response to pheromone in non-odorized air (Table 1). Similarly, heptanal 0.1% did affect neither the percentage of males that took off (χ^2^ = 0.0, df = 1, *p* = 1.0) nor the number of males that performed a partial flight, or those that reached the source (χ^2^ = 0.0868, df = 1, *p* = 0.7683).

**Table 1:** **Flight responses of virgin male ***A. ipsilon*** to the pheromone and a mixture of pheromone and heptanal at 0.1% (Series A) or 1% (B)**.

**Series**	**Stimulus**	**Number of males**	**Activation**	**Take-off**	**Partial flight**	**Source approach**
A	Pheromone blend	81	98.8	98.7	75.3	22.1
	Heptanal 0.1%	55	96.4	84.0	58.0	0.0
	Pheromone + heptanal 0.1%	55	100.0	98.0	74.0	26.0
	Control (MO)	54	88.9	81.6	51.0	0.0
B	Pheromone blend	75	100.0	97.3	72.0	16.0
	Heptanal 1%	76	97.4	88.2	63.2	1.3
	Pheromone + heptanal 1%	76	100.0	92.1	69.7	17.3
	Control (MO)	74	90.5	75.4	48.6	0.0

*Data are the percentages of four behavioral items carried out within 90 s*.

Significantly more males flew in presence of heptanal 1%, compared to the control (no odor; Take off: χ^2^ = 6.3574, df = 1, *p* = 0.0117; Partial flight: χ^2^ = 6.0639, df = 1, *p* = 0.0138), but almost no males (1.3%) approached the heptanal source. Heptanal at 1% did not affect the responses to the pheromone blend as shown by scores for: activation and take off (χ^2^ = 0.0, df = 1, *p* = 1.0), partial flight (χ^2^ = 0.0868, df = 1, *p* = 0.7683) or source approach (χ^2^ = 0, df = 1, *p* = 1).

**Figure 10:**
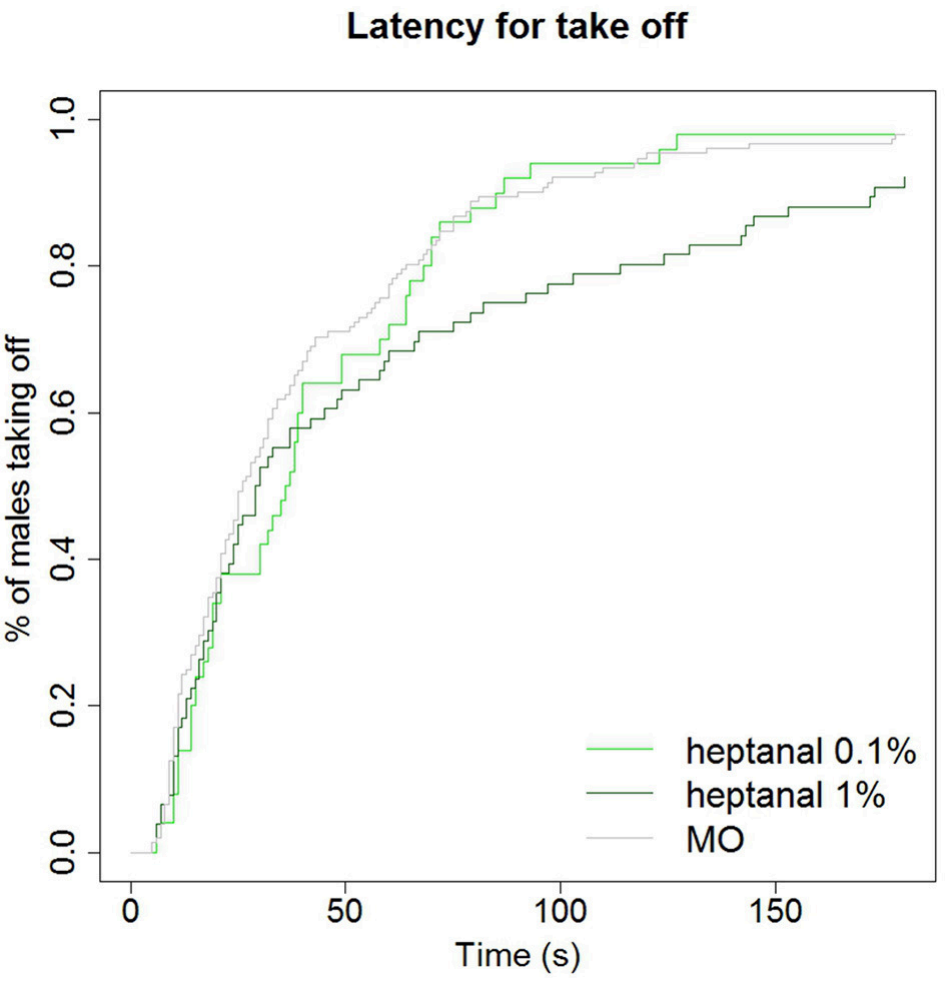
**Delay of take off in behavioral response to pheromone in different backgrounds**. A background of heptanal at 1% slows down the behavioral response of *A. ipsilon* males to the pheromone compared to a mineral oil or 0.1% heptanal-background. Kaplan Meier estimator curves showing the time at which the maximum firing frequency is reached in response to the 100 ng pheromone blend in different backgrounds: mineral oil (MO, *N* = 81), heptanal 0.1% (*N* = 53), and heptanal 1% (*N* = 36).

Although the same proportions of males responded to pheromone in a non-odorized background as in an heptanal background, the latency for take-off was significantly increased by the heptanal background (χ^2^ = 6.6 on 2 degrees of freedom, *p* = 0.037; Figure 10). Thus, the presence of a heptanal background resulted in a significant delay in their response.

## Discussion

We show in the current work that responses to sex pheromone in male *A. ipsilon* moths are modified throughout the first levels of the olfactory pathway by certain VPCs applied as background odors, depending on the concentration and the identity of the VPC and on the pheromone dose used for stimulation. Male moths orient, however, behaviorally as well toward the pheromone in a heptanal background as in a control background even though with a longer response delay. These experiments confirm our previous results, that heptanal activates the Phe-ORNs of *A. ipsilon* resulting in a sustained firing activity when presented as a sustained background stimulus. Accordingly, this effect on receptor neurons resulted in a calcium release in the macro-glomerular complex, thus confirming that the floral volatile heptanal is activating both the pheromone- and the general odorant- subsystems (Rouyar et al., 2015). Further, intracellular recordings indicated that central neurons within the MGC were activated by heptanal (Rouyar et al., 2015). As described previously, the level of activation produced by heptanal was generally lower compared to that of pheromone, indicating that heptanal is a partial agonist of the pheromone (Rouyar et al., 2015).

So far, binary interactions between odorants have been generally studied by stimulating the antennae simultaneously with the two odorants being released from a single source. In the present study we chose to apply heptanal as a long-lasting stimulus, starting before the pheromone pulse, to get closer to the temporal dynamics of interactions between odorants produced by different organisms in a natural context. *In natura*, pheromone and plant odors are released from spatially distinct sources with very different characteristics: the pheromone is emitted from a tiny gland (a point source) while VPCs are released from numerous inflorescences or large foliage masses creating a denser odor background. Thus, insects are exposed to higher concentrations, and for longer time periods to VPCs compared to pheromone. This protocol enabled us to investigate whether a good contrast (i.e., the relative difference between neural activity triggered by background and response to signal pulse) was maintained at the receptor neuron level and in the MGC in an odorized background. Antennal and central neurons maintained an increased level of firing activity during the entire time of presentation of heptanal, which should greatly affect further coding of an additional signal. Actually, when the pheromone was presented as a brief pulse during the stimulation with heptanal, to which neurons responded, the absolute response to the pheromone was reduced. This type of interaction between heptanal and pheromone seems very similar to a mixture suppression effect (For definitions see for instance Ache et al., 1988), the response to a blend of two active components being less than the response to the most active component. Furthermore, instead of considering absolute levels of firing, expressing the relative level of firing evoked by a pheromone pulse over the activity elicited by the sustained presentation of heptanal revealed a strong contrast decrease, the change in firing activity being barely detectable. The additive effect of heptanal and linalool when 100 ng of the pheromone was used for stimulation in calcium imaging experiments, contrasting with results of electrophysiological recordings, might be explained by the low temporal resolution of this method and variation in response delays between pheromone and VPC responses. Simultaneous stimulation with heptanal and pheromone had earlier revealed a suppressive effect within the MGC in calcium imaging experiments in *A. ipsilon* (Deisig et al., 2012).

The concept of contrast has been largely used in sensory sciences, for instance to describe how the visual system extracts shapes from a complex scene. Contrast has been comparatively less considered in olfaction, probably because the absence of spatiality of odors makes it more difficult to conceptualize odor-contrast compared to visual-contrast. However, analyzing the relative difference in perceived signal intensity would bring a better view of the mechanisms involved in extracting the signal from the background. Olfaction is considered as a highly integrative modality, most odors being aerial mixtures of chemical compounds. The capacity of the olfactory system to detect individual volatile components and to analyze activity from the different types of ORNs has been extensively studied because it is the basis for odor discrimination. An abundant literature has investigated mechanisms for blend coding, especially to understand the processes leading to the perception of a whole odor from the detection of its components. These studies have revealed the importance of interactions between blend constituents, for instance mixture suppression, synergy, or salience of one constituent over the other, in building the olfactory image of the blend. Similar interactions between chemicals must also take place during the extraction of a blend-odor shape from the background, an almost reverse process to blend coding. The mechanisms of extracting a behaviorally relevant compound or odor blend from a more or less complex background have recently been investigated in both insects and vertebrates. The recognition of nectar-bearing flower odors in the sphinx moth *Manduca sexta* for example depends highly on the presence of surrounding flower odor sources and the balance of excitation and inhibition in the antennal lobe modifies behavioral choices (Riffell et al., 2014). Also in the locust, using individual odor components, neural representation of a foreground stimulus was altered by a background. However, overlap of spatio-temporal activity patterns within the antennal lobe evoked by the same foreground stimulus in different backgrounds, allowed its recognition (Saha et al., 2013). A calcium imaging study in mice revealed that the capacity to detect individual odorants within variable backgrounds depends highly on the overlap in spatial representation of the odorants to be discriminated within the olfactory bulb (Rokni et al., 2014). Lateral inhibition, a mechanism known to contribute to contrast effects in vision, seems to play an important role also in the integration of olfactory input (Urban, 2002). Contrast enhancement seems to increase the capacity of the projection neurons of the MGC to follow stimulus intermittency since a GABA_A_ receptor antagonist, bicuculline, impedes the capacity of *Manduca sexta* antennal lobe neurons to follow intermittent patterns of pheromone stimulation (Lei et al., 2009). Such mechanisms might be critical to maintain signal discrimination performances in a rich olfactory environment. Our finding that a heptanal background also reduces the relative response to pheromone pulses over the background pleads for more detailed investigation of contrast enhancement effects.

The responses to heptanal activity observed in the MGC may result either from a direct activation of the pheromone sub-system through the afferent Phe-ORNs, or alternatively from an inter-glomeruli redistribution of the activity generated in the general odorant neurons by plant volatile compounds. Heptanal stimulates receptor neurons tuned to general odorants (Rouyar et al., 2015) in the antenna, which project into general odorant glomeruli. This input could be redistributed toward pheromone specific areas of the AL by local interneurons that connect the MGC with the general odorant glomeruli (Hansson and Anton, 2000). Linalool, which also stimulated Phe-ORNs interacted with pheromone perception at the level of central neurons in a similar way. However, α-pinene, which did not stimulate the Phe-ORNs specifically tuned to Z7-12:OAc (Munoz, unpublished data) did not. In *Cydia pomonella*, apple tree volatiles alone did not elicit any Ca^2+^ signal in the MGC while blends of codlemone (pheromone) plus plant volatiles produced a clear synergistic response (Trona et al., 2013). This suggests that the effects in ALs of the heptanal and linalool backgrounds we observed are mainly due to peripheral interactions and principally to the activation of pheromone receptor neurons by heptanal.

In the wind tunnel, male *A. ipsilon* performed as well in non-odorized air as in heptanal-odorized air, indicating that they were able to orient toward a pheromone source in spite of negative sensory effects of heptanal. However, male moths took flight significantly later in response to pheromone in the presence of heptanal. Interestingly, a delay in response to pheromone was also observed at MGC neuron level in the presence of heptanal. The masking effects observed at two levels of the pheromone sub-system, the afferent neurons and the MGC, decreased pheromone sensitivity and caused a response delay, but once triggered, the final response was relatively independent from the background. Alternatively, a higher dilution rate of heptanal in the wind tunnel may also account for the difference between sensory and behavioral effects.

The experiments presented in this paper demonstrate that an odorant background of individual VPCs may affect the perception of a specific signal resulting in some alteration of the behavior. A recent study did not reveal changes in behavioral pheromone responses in a wind tunnel in presence of the head-space of a single plant, concluding that natural emissions are too low, compared to concentrations used in laboratory studies, to alter pheromone detection (Badeke et al., 2016). However, in natural conditions insects are exposed during long periods of time to very rich odorscapes from which they must extract ecologically relevant signals, and interference of individual compounds and complex plant odors with pheromone signals might also be different. Therefore, it is very likely that interference between VPCs and intraspecific signals occurs even under natural conditions. It will be important in the future to study the influence of odor backgrounds constituted of several VPCs on pheromone blend recognition in male moths, in order to understand adaptive mechanisms of species recognition in a complex plant environment.

## Ethics statement

All the experiments described in the manuscript were performed with laboratory-reared insects. No special permit was required. After experiments, moths were quickly killed by freezing.

## Author contributions

SA, FD, ND, DL, MR, and AR together conceived and designed the study. FD, ND and AR planned and carried out imaging and electrophysiological experiments. DL, MW, and TB carried out wind tunnel experiments. SA, FD, ND, AR, and MR analyzed and interpreted the results, prepared the figures and wrote the paper. All authors critically revised the article.

### Conflict of interest statement

The authors declare that the research was conducted in the absence of any commercial or financial relationships that could be construed as a potential conflict of interest.
